# A Plug‐and‐Play Volume Minimizing Micromixer

**DOI:** 10.1002/advs.202510268

**Published:** 2026-02-27

**Authors:** Kirill Kolesnik, Philipp Segeritz, Daniel J. Scott, David J. Collins

**Affiliations:** ^1^ Department of Biomedical Engineering The University of Melbourne Melbourne Victoria Australia; ^2^ The Florey Institute The University of Melbourne Parkville Victoria Australia; ^3^ Department of Biochemistry and Pharmacology The University of Melbourne Parkville Victoria Australia; ^4^ Grame Clarke Institute The University of Melbourne Parkville Victoria Australia

**Keywords:** 3D printing, lab‐on‐a‐chip, microfluidics, micromixer

## Abstract

Microscale fluid mixing has numerous applications where rapid and efficient mixing is required, including drug discovery, bio‐analysis and point‐of‐care diagnostics. Conventional soft lithography processes, however, make the integration of robust and reliable mixing difficult to implement in practice, especially across different flow rates. Here a 3D‐printed plug‐in micromixer designed for rapid and modular integration with microfluidic devices that addresses long‐standing integration challenges for micromixing in microfluidic devices is presented. The micromixer is based on the split‐and‐recombine (SAR) channel topology employing an optimized 3D geometry, minimizing internal volume and fluidic resistance. A micromixer with 60‐µm internal channels is fabricated and experimentally tested, demonstrating efficient mixing while maintaining a reliable seal with a PDMS microfluidic device. Accordingly, the modular micromixing device enhances the efficiency and usability of microfluidic systems, offering a promising solution for biomedical and analytical applications. This conceptually elegant plug‐in approach represents a unique advance in the practical integration of 3D printed functionality with microfluidics devices.

## Graphical abstract

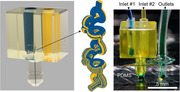

## 1 Introduction

Efficient and rapid mixing is essential for many microfluidic applications in biomedicine and chemical analysis. For instance, rapid and thorough micromixing is critical for chemical synthesis [1], immunoassays [2], drug screening [3], protein engineering [4], and nanoparticle formation [5, 6]. For example, rapid mixing is key in the co‐solvent method for copolymeric nanoparticle assembly because it directly controls the early stages of particle formation, where mixing rate determines the size, morphology, and uniformity of the nanoparticles [7, 8]. Other instances where the speed, completeness, and immediate proximity of mixing to downstream analysis is vital to the desired outcomes spans many applications, including chemical/biological reactions [9], microgel manufacture [10], protein folding [11], and calcium signaling [12], where pre‐mixing of bulk solutions otherwise yields outcomes that may be non‐desired or difficult to control compared to a micromixing strategy. To accurately control this mixing, especially for small sample quantities, micromixers can be used to mix two or more fluids. Since the characteristic dimensions of such microfluidic systems typically range from tens to several hundred micrometers, they operate predominantly in the laminar flow regime. In such conditions, however, fluid mixing primarily relies on molecular diffusion, which is inherently slow [13]. To address this, both active and passive micromixers approaches have been developed to enhance mixing rates. Active micromixers utilize external excitations such as ultrasonic waves [6, 14], electroosmotic forces [15], dielectrophoresis [16], electrohydrodynamics [17], and magnetic fields [18]. While these approaches significantly improve mixing rates, they introduce significant system complexity, fabrication cost, heat generation, and incompatibility with many biological and delicate samples [19].

In contrast, passive micromixers rely solely on hydrodynamic and/or diffusive effects to enhance mixing, eliminating the need for external forces. At relatively high Reynolds numbers (Re), the introduction of obstacles [20], or channel arrangements such as Tesla valve and herringbone mixers [21, 22] leverage advective effects to enhance mixing, though these have the disadvantage of requiring specific ranges of flow rates within to operate effectively, significantly limiting their integration with microfluidic devices operating at lower Re [23]. Conversely, split‐and‐recombine (SAR) micromixers can robustly achieve efficient mixing through repetitive fluid folding, increasing interfacial contact between fluid layers and reducing diffusion lengths in a manner that is largely decoupled from the fluid flow rate, enabling reliable mixing even at low Re [24, 25, 26]. However, their practical use is limited by the need for complex three‐dimensional structures, as the fabrication methods required may be incompatible with, or add complexity to, conventional microfluidic implementations based on soft lithography or injection moulding [27]. This mirrors the issues commonly associated with the use of micromixers generally. For instance, micromixers may be fabricated directly on the same chip as the primary microfluidic channel [2, 28], but this approach increases device footprint, fabrication complexity, and cost, and may lead to suboptimal mixing if the target mixing structure is not optimized for the flow rates involved. Moreover, fabrication constraints limit compatibility with other microfluidic geometries in the first instance, necessitating the use of planar mixing channels that are less effective than three‐dimensional ones [29]. Due to these difficulties, the integration of micromixer approaches with other microfluidic technologies is often overlooked, preventing their widespread adoption.

Examining the microfluidic devices themselves, recent advancements in 3D printing make it an attractive approach for microfluidic device fabrication due to its versatility, affordability, and precision [30]. 3D printing has the potential to improve the modularity of microfluidic components, this being long recognized as a high‐value proposition to improve the effectiveness of lab‐on‐a‐chip systems [31, 32, 33, 34, 35]. However, high‐resolution 3D printing is inherently slow, necessitating compact designs to reduce printing time and improve throughput. Additionally, high‐resolution printers have limited build plate sizes, which constrain the overall print dimensions and impose restrictions on the design of microfluidic chips, including the integration of fluidic ports. Moreover, aligning and bonding such printed channels with electrode or other structures on a planar surface is non‐trivial. Further, while threaded connectors provide reliable sealing, they are bulky and may not be suitable for compact microfluidic systems [36]. A further challenge is the inherent surface roughness of 3D‐printed components, which can affect the reliability of fluidic connections. Accordingly, while 3D printed channels are a topic of increasing investigation, the bulk of experimental and application‐based microfluidic implementations continue to rely on more conventional and planar fabrication approaches, in which the integration of robust micromixing is challenging.

**FIGURE 1:**
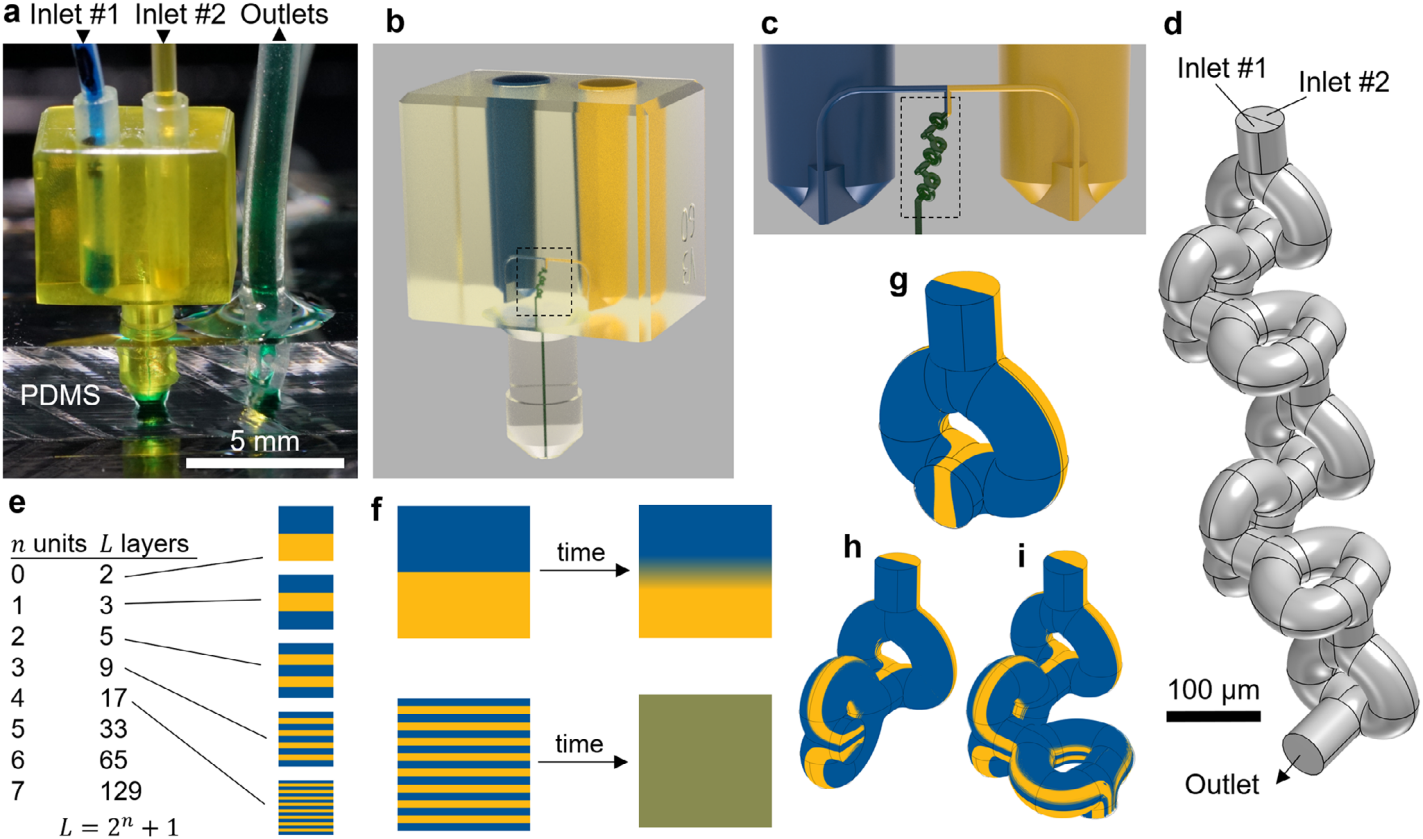
3D‐printed plug‐in SAR micromixer. (a) 3D printed micromixer inserted into a PDMS microfluidic device. (b) Mixing device 3D render. (c) Close‐up view of the fluid path within the micromixer. (d) The micromixer core including 6 SAR units. (e) Theoretical representation of fluid layering as it progresses through *n* mixing units. (f) Schematic representation of concentration profiles in the T‐mixer and the laminating mixer, demonstrating diffusion‐induced mixing over equivalent time. (g) One (h) two and (i) three mixing units.

In this study, we introduce a modular, plug‐in SAR micromixer approach designed for seamless interfacing with conventional microfluidic devices, e.g. those made of PDMS (Figure 1). Our SAR micromixer utilizes a unique 3D curvilinear channel geometry that is made possible via high‐resolution 3D printing to minimize internal volume and fluidic resistance while maintaining high mixing efficiency, retaining the fundamental SAR topology while reducing path lengths, improving performance. We employ digital light processing (DLP) 3D printing to fabricate our micromixer with internal 60 µm diameter channels in total comprising extremely small, nanoliter‐scale internal volumes, minimizing dead volume and waste. Computational fluid dynamics (CFD) simulations and experimental validation are utilized to evaluate mixing performance. Our plug‐in design enables seamless integration with multiple microfluidic devices, facilitating easy connection, operation and removal without leakage. This feature allows the micromixer to be reused across multiple disposable chips, addressing key challenges in modular microfluidic system integration. Additionally, the micromixer features tapered inlets, allowing compatibility with standard off‐the‐shelf tubing. This design ensures ease of use, eliminating the need for specialized equipment and facilitating widespread adoption in microfluidic applications.

## 2 Results and Discussion

### 2.1 Micromixer Design

The micromixer is integrated into a 3D‐printed device, which features two tapered fluidic inlets, each 4.75 mm in length with a 1‐degree taper (see Figure 1a; Figure S1). During assembly, a section of compliant silicone tubing is first inserted into the inlet, followed by a PTFE tube. The silicone tubing acts as a gasket, ensuring a secure and leak‐resistant connection. The incoming fluids from both inlets pass through a micromixing core manifold before exiting via a barbed outlet pin (Figure 1b,c). This pin, 3.5 mm in length and 1.8 mm in diameter at its widest point is designed to securely interface with a PDMS device featuring an inlet approximately 1.5 mm in diameter and 1.5 mm deep. Details of the micromixer assembly and Computer‐aided design (CAD) files are provided in the Supporting Information (Movie S1 and design files).

The SAR approach is predicated on the use of repeated mixing units that split, rotate and recombine the flow, increasing the number of fluid layers, and decreasing the distance between layers, with subsequent mixing units. The fundamental structure of the micromixer is depicted in Figure 1d. It comprises seven identical units (Figure 1e), each rotated 90 degrees relative to the preceding one. Each unit functions by splitting and recombining the fluid stream such that the input of one mixing unit is replicated twice in its output, enabling a repetitive folding of fluid layers (see Figure 1f). This configuration significantly reduces the molecular diffusion path length, thereby enhancing the mixing efficiency. This split‐and‐recombine design facilitates efficient fluid layering, where the number of fluid interfaces *I_n_*, number of alternating fluid layers *L_n_*, and average fluid layer thickness δ_*n*_ relates to the number of mixing units *n*, with

(1) $$\;I_{n}=2^{n}\;\;L_{n}=2^{n}+1\;\delta_{n}=\frac{\pi D}{4L_{n}}\;,$$

where, for δ_*n*_, *D* is the channel diameter, and assuming that each fluid lamella has a given area *A*/*L_n_* (with *A*  =  π*D* ^2^/4 for a circular cross‐section), and with the condition that the initial input (at n = 0) consists of two inlet streams separated by one fluid‐fluid interface. A notable detail is that the fluid layer dimensions will vary locally along the flow direction as the flow transitions to fully developed parabolic flow within each channel section. Here the hydrodynamic entrance length is given by ℓe≈0.05ReD (for laminar flow), and the fluid flow is ‘reset’ between mixing units with the remapping of streamlines. Given typical microfluidic Re = 0.1–100, this equates to entrance lengths that can be either smaller or larger than the path length for a given mixing unit bifurcation (approx. 3*D*), though ultimately the mixing enhancement is a function of the number of adjoining fluid layers, with (in examining a parabolic or transitional flow profile in the channel cross section) a local increase in layer thickness near a channel wall being partially mitigated by the longer residence time for the fluid in a lower flow velocity. The actual distribution and dimensions of distinct fluid lamella across any given channel cross section will accordingly be a function of the Reynolds number, as examined in Figure S2, though nevertheless with the impact that the number of fluid layers (and accordingly the degree and rapidity of mixing) will scale with ∼2^*n*^; with the use of seven mixing units, for instance, this results in the generation of 129 layers. Importantly, decreasing the lamellar thickness decreases the diffusion time, where the characteristic 1D diffusion equation results in a mixing time $t_{mix}\;∼\;\delta_{n}^{2}/D_{d}$ scaling, where *D_d_* is the diffusion constant, and where the fluid layer thickness scales with 1/2^*n*^ and the mixing rate accordingly scales with $\frac{1}{t_{mix}}∼4^{n}$. That is, reducing the layer thickness by 10x (i.e. with 20 fluid layers instead of 2) results in 100x faster diffusion, and with 129 layers from 7 mixing this results in a ∼4000x decrease in diffusive mixing time (compared to two initial fluid layers).

The internal diameter of the micromixer channels are 60 µm, resulting in a total 7.7 nL internal volume within the mixing manifold; while internal volume in other micromixing geometries is not typically reported, this represents a miniscule residual dead volume compared to micromixing approaches relying on larger channel dimensions and/or other (non split‐and‐recombine) mixing strategies, and is orders of magnitude smaller than the volume contained in any tubing leading to the micromixing manifold itself. The barbed outlet pin also maintains a 60 µm internal channel to minimize dead volume and efficiently deliver the mixed solution into the receiving microfluidic channel. Giving the scalability of this approach, where an arbitrary number of mixing units can be implemented with minimal changes in internal volume (e.g. four units corresponds to ∼3.5 µm fluid layers in a 60 µm channel, seven units to ∼500 nm fluid layers, and ten units to ∼5 nm ones), effective mixing can be achieved essentially regardless of characteristics such as flow rates and diffusion constants. This direct mixing‐to‐delivery configuration thus reduces the time between mixing and downstream analysis, especially compared to approaches based on flow‐dependent chaotic advection, which is critical for time‐sensitive applications.

### 2.2 Numerical Modelling

To assess the evolution of fluid layers across mixing units, we performed computational modelling of the fluid flow within the mixing core using COMSOL Multiphysics. To demonstrate the effective performance across flow velocities, in the implemented simulations we use volumetric flow rates of *Q* = 1, 10, and 100 µl min^−1^. This corresponds to the average fluid velocity within the 60 µm wide channel of 6, 59, and 589 mm s^−1^, and Reynolds numbers of 0.4, 4, and 40. At a moderate flow rate of 10 µl min^−1^, the mixing core is modelled to induce a pressure drop of Δ*P*  =  526 Pa, and the corresponding hydraulic resistance is *R*  = Δ*P*/*Q*  =  3.2 · 10^12^ Pa·s/m^3^.

**FIGURE 2:**
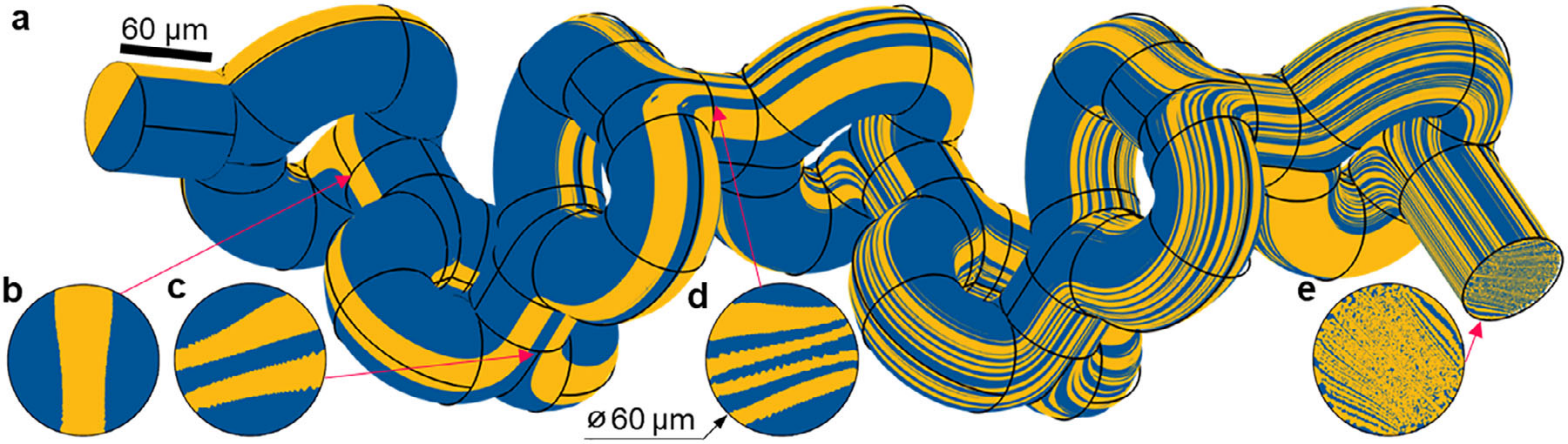
CFD modelling of fluid flow at *Re* = 4 in our 3D‐printable micromixer core. (a) Fluid flow streamlines in the microchannel. (b–e). Progressive fluid lamination after passing through 1, 2, 3, and 7 mixing subunits.

Figure 2a plots the fluid streamlines at the flow rate of *Q* = 10 µl min^−1^ (*Re* = 4) which visualizes the fluid pathways within the manifold, showing the laminar flow of fluid components. The fluidic distribution across key cross‐sections (Figure 2b–e) demonstrates that each mixing unit essentially doubles the number of fluid layers. A lower flow rate (*Q* = 1 µl min^−1^) produces an essentially identical flow distribution (see Figure S2a,b). This indicates that at lower *Re* values, despite the dominance of viscous effects which act to impede advective mixing, the micromixer nevertheless reliably produces far shorter diffusion path length scales. At a higher flow rate (*Q* = 100 µl min^−1^, *Re* = 40), the flow profile exhibits more pronounced inertial effects that reduce the laminar nature of the flow, producing somewhat less uniform mixing (Figure S2c). This demonstrates the superior micromixer performance across a wide range of Reynolds numbers, particularly at lower Reynolds numbers, addressing the poor low‐Re laminar regime of advection‐based mixers.

### 2.3 Experimental Validation

**FIGURE 3:**
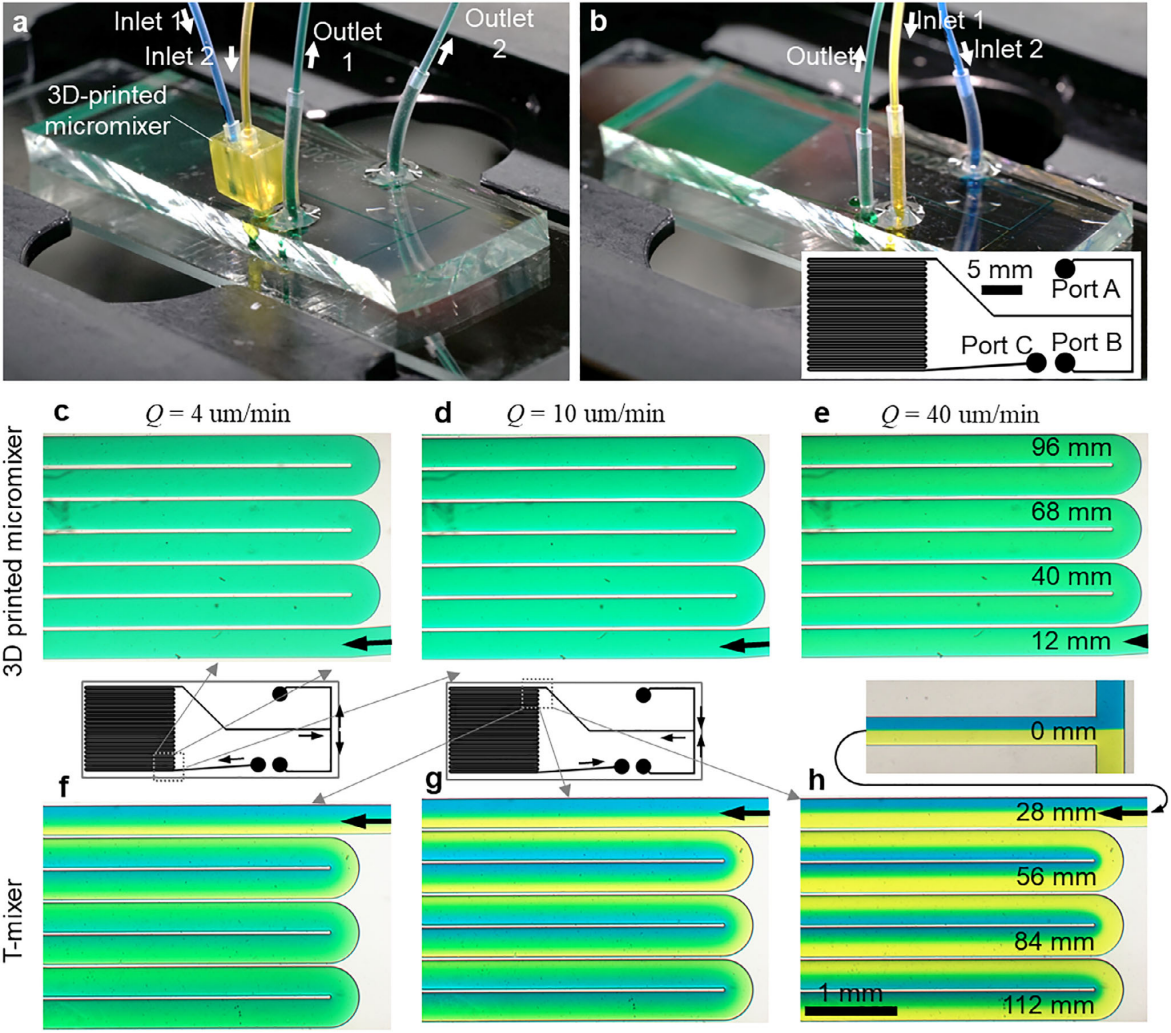
Experimental validation of the 3D‐printed micromixer. (a) 3D‐printed micromixer plugged into Port C of the PDMS microfluidic serpentine device, where Ports A and B serve as outputs. (b) Comparative fluid mixing in the T‐mixer, where two fluids are injected through Ports A and B. Mixing tests with coloured fluids: (c–e) Microscopy images of the fluid flow in the PDMS serpentine microchannel after passing through a 3D‐printed micromixer, demonstrating equivalent homogeneous mixing at different flow rates. (f) The T‐mixer junction with two coloured liquids. (g–i) Microscopy images of the T‐mixer performance, with performance deteriorating at higher flow rates. Arrows from serpentine device layouts indicate the corresponding areas of the microfluidic device for each set of images. (c), (f) Flow rate *Q* = 4 µL min^−1^; (d), (g) *Q* = 10 µL min^−1^; (e), (h) *Q* = 40 µL min^−1^. In (e,h) the total distance travelled by the fluid in the PDMS channel is indicated.

The mixing performance was experimentally validated by introducing colored fluids into the micromixer and analyzing the downstream flow within a PDMS serpentine channel (Figure 3a). The PDMS device features a serpentine channel with two inlet/outlet channels at one end which allows us to compare the mixing performance of the plug‐in device with a conventional T‐mixer (Figure 3b). The mixing device was inserted into a 1.5 mm punched hole in the PDMS channel layer. As the hole diameter was smaller than the 1.8 mm barb fitting, a leak‐proof interface was achieved. The micromixer exhibited robust performance, with no leakage observed over multiple plug‐in cycles. The same microfluidic device was operated in reverse to evaluate the T‐mixer performance for comparison. The fluidic connectors were further tested using a pressure pump and demonstrated the ability to withstand pressures of up to 2 bar (the maximum our pressure driven system could supply) without leakage.

The plug‐in SAR micromixer achieved a uniform color at the micromixer output (Figure 3c–e), with the fluid being fully mixed upon entering the channel irrespective of the fluid flow rate utilized. In contrast, the conventional T‐mixer, tested under identical conditions, exhibited incomplete mixing within the serpentine channel, particularly at higher flow rates, with diffusing mixing occurring along the serpentine channel length (Figure 3f‐h). Achieving a similar level of mixing with a T‐mixer requires substantially longer channel lengths, e.g. centimeters–meters depending on the flow rate (compared to the total ∼1 mm micromixer path length), vastly increasing the overall device footprint and fluidic resistance. Accordingly, the split‐and‐recombine architecture of the plug‐in micromixer facilitates rapid and efficient mixing within a compact design, making it particularly advantageous for applications requiring miniaturization and high‐performance fluid handling.

## 3 Discussion

### 3.1 Split‐and‐Recombine Mechanism

The operating principle of a SAR micromixer is based on the recursive division and recombination of fluid streams, which exponentially increases the interfacial area between them. Each SAR unit splits the incoming fluid into two or more substreams and then geometrically rearranges them before merging, facilitating the lamination of distinct fluid layers. Each mixing unit essentially doubles the number of fluid layers according to Equation 1, halving the characteristic diffusion path with each unit. Here mixing time τ scales quadratically with the characteristic diffusion length and is inversely proportional to the molecular diffusivity *D*, highlighting the significance of diffusion path shortening for mixing improvement. Accordingly, if the separation distance between fluid components is reduced by an order of magnitude relative to the initial state, the characteristic diffusion‐driven mixing time is reduced by approximately two orders of magnitude. In the case of a 6 SAR micromixer producing 65 interleaved fluid layers, for instance, this results in a ∼1000x reduction in mixing time, compared to a 4000x reduction for 7 SAR micromixer. While the choice of SAR units can be tuned, if needed, as a function of the desired mixing rate and flow lamella width for a given target application, in all such cases SAR devices have the effect of massively enhancing the bulk‐scale concentration uniformity across the cross‐section (compared to 2 layers).

### 3.2 Plug‐in Design

The modular configuration of the micromixer, permitting this to be used with any PDMS device requiring pre‐mixed input, is a key advantage compared to integrating this difficult‐to‐manufacture functionality on‐chip, especially for single‐use microfluidic devices (e.g. containing analytes or detection zones). Further, there are advantages in the use of such a plug‐in compared to an entirely 3D printed device, as incorporating electrodes, detection zones, surface coatings and other functionality remains ideally suited toward planar fabrication techniques. Moreover, 3D printed materials often have limited optical transparency, and are limited by the inherent fluorescence of photocurable polymer in florescence microscopy. To address these limitations, our hybrid device leverages the advantages of both approaches, where 3D printing is used to realize complex volumetric geometries essential for efficient micromixing, while soft lithography can be employed to realize the wide variety of microfluidic architectures that have already proven their utility across a broad spectrum of scientific literature and commercial applications.

### 3.3 Design Optimization

The internal volume is an essential parameter in micromixer design, as it directly influences the mixing time at a given flow rate and sample wastage. Despite its importance, internal volume is rarely reported in the literature for micromixer designs, complicating performance benchmarking across devices. To reduce sample wastage and minimize channel residence time, our design incorporates shortened fluidic connectors within each mixing unit, the small channel diameters, the curvilinear paths, and circular cross‐sections. This collectively represents a highly volume‐minimized split‐and‐recombine (SAR) topology comprising just several nanoliters in the mixing region leading to the device outlet, serving to also minimize the time between mixing and administration, highly important for time‐sensitive reactions. The use of circular cross‐sections instead of a square further offers several advantages relevant to performance and fabrication. First, circular channels exhibit lower hydraulic resistance for a given cross‐section area, reducing pressure drop across the micromixer. Second, the absence of sharp corners in circular geometries improves strain rate uniformity, beneficial for maintaining cell viability in biological applications. Third, circular channels are better suited to 3D printing as they have smoother overhangs, essential for high‐quality prints.

### 3.4 Scaling and Fabrication

While scaling down the micromixer geometry reduces its internal volume and enhances mixing efficiency, such miniaturization is typically constrained by fabrication limitations, and may also lead to deteriorated performance in certain applications. For instance, channel clogging, especially with biological samples such as ∼10 µm cells, is an increasing factor with smaller channel dimensions [37, 38]. While other fabrication methods might nevertheless reduce micromixer dimensions beyond the 60 µm channel diameters implemented here (with DLP), their fabrication and utilization may also not be as practical. For example, two‐photon polymerization (2PP) enables the fabrication of complex 3D microstructures with sub‐micron resolution, allowing for channel diameters on the order of ∼1 µm. While such miniaturization could reduce diffusion path lengths, in practice high‐resolution 2PP approaches tend to be inappropriate for manufacturing larger scale (i.e. ∼1 cm) parts, necessary for easy handling and integration with microfluidic channels at practical printing speeds and costs. Further, while other 3D manufacture approaches such as laser micromachining [39, 40] and multi‐level microchannel bonding approaches [41, 42] may be able to realize similar channel dimensions as those used in this work, such techniques add significant complexity to the manufacturing process, and are unable, without further processing, to readily define the exterior geometry that is necessary to replicate the plug‐and‐play nature of our DLP‐printed design. Recent DLP‐based microprinting approaches [43] have the potential to further reduce channel volumes while retaining the overall plug‐and‐play design dimensions, though at the expense of increased clogging risk for cell‐related applications. While ownership of high‐end printers remains costly, printing‐as‐a‐service is emerging as a promising option to reduce expenses, including (at present) the vendors of the system utilized (BMF, USA), third party vendors and academic fabrication facilities, where an increasing range of printers offer resolutions capable of generating ≲ 100 µm enclosed channels. Moreover, the Anycubic Photon Mono 4 10K resin printer, released in 2025, offers a 17 µm pixel resolution at a price < $300 USD, demonstrating the lowering barriers for high‐resolution fabrication. Accordingly, this technology is becoming increasingly affordable and accessible, supporting wider and more rapid adoption for 3D printed microfluidic components.

## 4 Conclusions

While micromixing approaches have been extensively examined, their widespread implementation has been limited by the poor performance of micromixers and/or significant difficulties in their integration. Here we present a split‐and‐recombine (SAR) micromixer that enables practical and modular use with microfluidic systems. By leveraging DLP‐based 3D printing, we demonstrate a compact and efficient mixing device with microscale internal channels (60 µm). The optimized SAR topology enhances mixing efficiency while minimizing dead volume (7.7 nL for a seven‐unit implementation) and fluidic resistance (3.2 · 10^12^ Pa·s/m^3^.), addressing critical limitations of previous passive micromixers. Experimental validation confirms that our micromixer reliably achieves effective mixing while maintaining a robust seal with PDMS‐based microfluidic devices. Its modular plug‐in design allows for easy reuse across multiple systems, eliminating the need for on‐chip fabrication and reducing both cost and complexity in microfluidic device development. Furthermore, the integration of tapered inlets enables seamless compatibility with standard tubing, enhancing usability without additional equipment. By overcoming key fabrication and integration challenges, this design expands the practical application of SAR micromixers. Our micromixer design can be further miniaturized to reduce dead volume and fabrication costs, subject to 3D fabrication resolutions. Moreover, our plug‐in concept presents a compelling approach for the integration of 3D printed elements with microfluidic systems generally, enabling the addition of further functionality to conventional microfluidic devices.

## 5 Methods

### 5.1 CFD Simulation

At low Reynolds numbers (Re), microfluidic flows are dominated by viscous forces, resulting in laminar, steady‐state behavior. The fluid was modelled as Newtonian and incompressible, with gravitational effects neglected. Consequently, the governing equations reduce to the continuity equation and the Navier–Stokes equations, expressed as

(2) $$\begin{array}{ccc} & & \rho_{f}\;\left(\frac{\partial u}{\partial t}+\left(u\cdot \nabla \right)u\right)=-\nabla p+\nabla \cdot \mu_{f}\left(\nabla u+{\left(\nabla u\right)}^{T}\right),\\ & & \rho_{f}\nabla \cdot u\;=0, \end{array}$$

where ***u*** denotes fluid velocity, *p* is pressure, ρ_*f*_ is density, and μ_*f*_ is the dynamic viscosity. The characteristic Reynolds number is given by

(3) Re=ρfuDμf,

where *D* represents the characteristic length scale of the system. The fluid assumed to have properties of Water at room temperature ρ_*f*_ =  998.2 kg m^−3^, μ_*f*_ = 1.003 · 10^−3^.

CFD simulations were conducted using COMSOL Multiphysics 6.1 within the Laminar Flow module. The finite element method (FEM) was employed with an unstructured tetrahedral mesh and boundary layers for accuracy. A no‐slip (the Dirichlet) condition was applied at the channel walls

(4) $$u\;\left(x,\;t\right)=0\;for\;x\in \Gamma ,$$

where Γ is the wall surface. A constant volumetric flow rate *Q* was imposed at the inlet, with

(5) $$Q=\int_{A}u\cdot ndS\;,$$

where *A* denotes the inlet cross‐section and **𝑛** is the inward unit normal vector. For the fully developed flow, the tangential flow component on the boundary is zero, with

(6) $$u-\left(u\cdot n\right)n\;=0.$$

Although the simulation was performed with a single‐phase flow at total rate 𝑄, streamlines were post‐processed separately from each half of the inlet, where each half assigned an effective flow rate of 𝑄/2. Streamlines of two fluids with equivalent properties are used to study fluid mixing within the micromixer. At the outlet, a zero‐pressure boundary condition was applied. The GMRES (Generalized Minimal Residual) iterative algorithm was used to solve the system of equations, having residual tolerance of 0.01. The iterative solver converged within 4 steps, with a total solution time of 105 s on a workstation with 32 GB RAM and an Intel Xeon processor. Peak physical memory usage was 10.65 GB.

The computed flow fields were analyzed to assess the micromixer's performance. Mixing efficiency was quantified by tracing 10^5^ fluid streamlines, providing a detailed representation of flow patterns. A Python‐based image analysis script was used to segment and plot streamline distributions in given cross sections.

To ensure mesh convergence, a refinement study following Devendran et al. [44] was performed, using the convergence function:

(7) $$C\;\left(g\right)=\sqrt{\frac{\int {\left(u-u_{\mathrm{ref}}\right)}^{2}\mathrm{dx}\;\mathrm{dy}}{\int {\left(u_{\mathrm{ref}}\right)}^{2}\mathrm{dx}\;\mathrm{dy}}}\;,$$

where *u* is the current solution, *u_ref_* is the reference solution where *d*_*mesh* *ref*_ =  1.5 µm. The flow was modelled at *Q* = 10 µl min^−1^. A 4 µm element size was selected, as it maintained accuracy while keeping *C*(*g*) < 0.005 (Figure S3). The resulting finite element model contained 2 354 063 elements, corresponding to 2 047 114 degrees of freedom.

### 5.2 Experimental Study

The micromixer was fabricated using a high‐resolution digital light processing (DLP) 3D printer (BMF, USA), capable of producing internal channels with diameters as small as 60 µm. After printing, the structures were cleaned in isopropanol to remove residual uncured resin, followed by UV post‐curing for 10 min to enhance mechanical stability and surface hardness.

For integration testing, polydimethylsiloxane (PDMS) microfluidic devices were fabricated via soft lithography. A negative photoresist SU‐8 3010 was spin‐coated on a 4‐inch silicon wafer. Microfluidic features were patterned using Karl Suss MA6 UV Mask Aligner (SUSS MicroTec, Garching, Germany) using a photomask with the microchannel design. PDMS (Sylgard 184) was then mixed with curing agent (10:1 elastomer/curing agent ratio) and cast on the wafer with patterned channels and cured in 80°C oven. The resulting PDMS layer contained channels 10 µm in height and 300 µm in width. Following curing, the PDMS part was bonded to a glass substrate using oxygen plasma treatment. Inlet and outlet ports were created with a 1.5 mm biopsy punch, and the micromixer was inserted directly into these ports. Silicone tubing (AWG30 PTFE, Masterflex, USA) were secured with UV‐curable adhesive (NOA adhesive, Norland Products Inc., USA) to establish robust fluidic connections. In experimental study, fluid flow was driven by a Gemini 88 Plus Dual‐Rate syringe pump (KD Scientific, USA), which controlled precise flow rates. Two 1 mL syringes were used to inject dyed fluids at equal flow rates. Commercial food dyes (Queen Fine Foods, Australia) were selected to approximate water‐like fluid properties. The mixing process was observed under a fluorescence microscope (BX51, Olympus, Japan). The fluidic connector sealing was tested using a pressure pump (Flow EZ, Flyigent, France), which has a pressure limit of 2 bar.

## Conflicts of Interest

The authors declare no conflicts of interest.

## Supporting information




**Supporting File 1**: advs74647‐sup‐0001‐SuppMat.docx.

**Supporting File 2**: advs74647‐sup‐0002‐MovieS1.mp4.

## Data Availability

The data that support the findings of this study are available in the supplementary material of this article.
