# Supplementary material for: A Plug‐and‐Play Volume Minimizing Micromixer
Source: Adv Sci (Weinh). 2026 Feb 27;13(27):e10268. doi: 10.1002/advs.202510268 (PMC13170216; doi:10.1002/advs.202510268)

**A plug-and-play volume minimizing micromixer**

**Supplementary information**


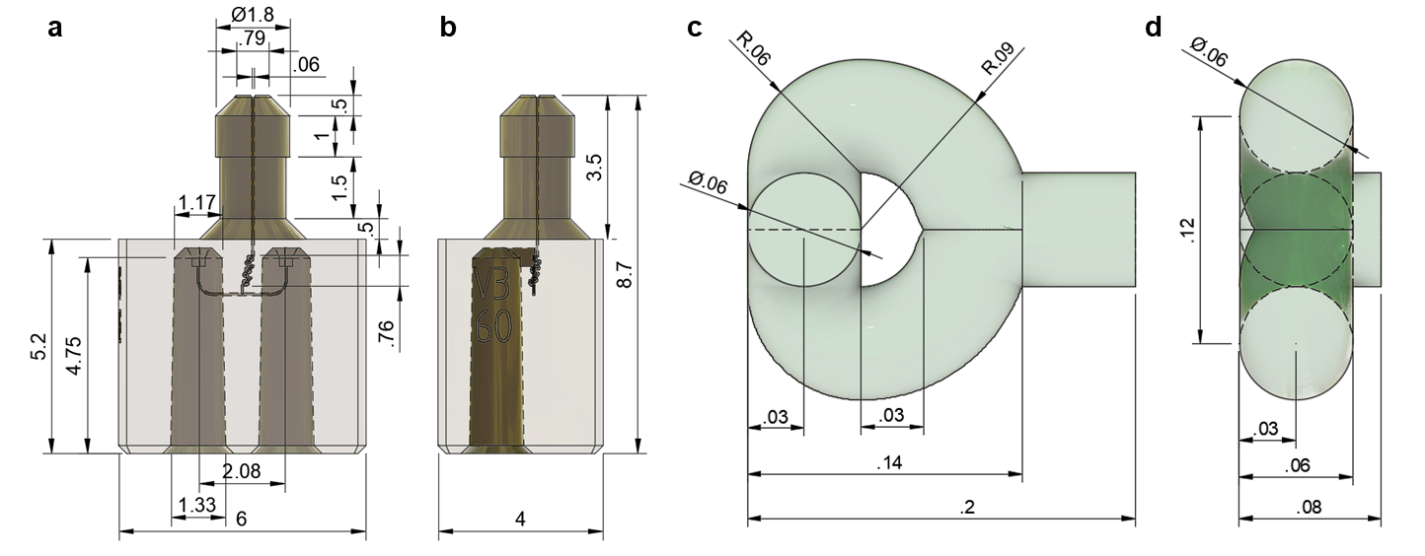


**Fig. S1**: Design views with dimensions of (a), (b) the 3D-printed micromixer and (c), (d) the mixing unit.


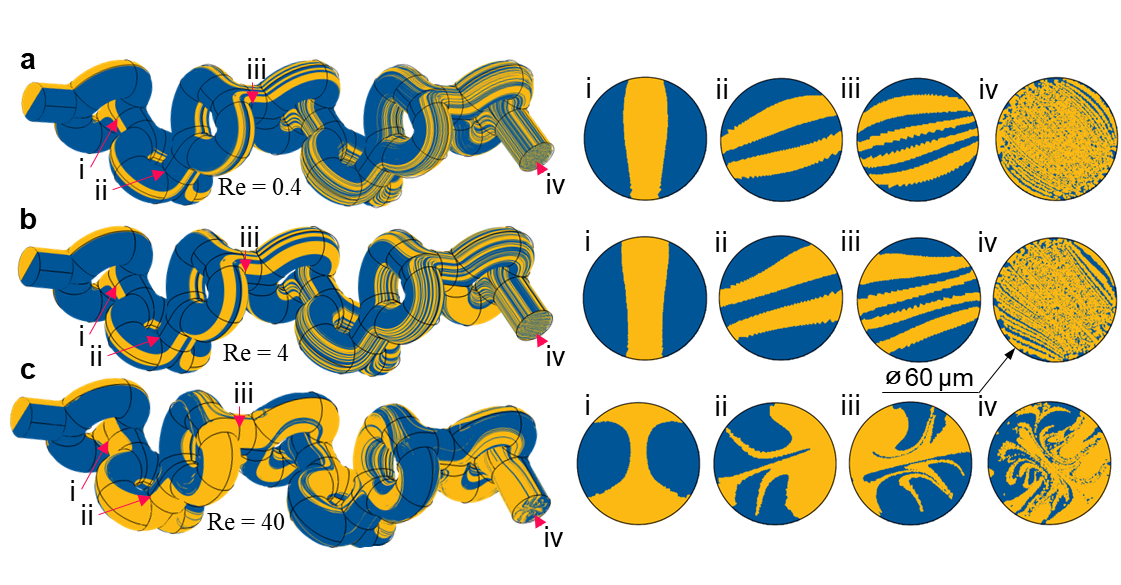


**Fig. S2:** CFD modelling of fluid flow at three flow rates: 1, 10, and 100 ul/min (a-c) corresponding to Reynolds numbers of 0.4, 4 and 40, respectively. Subfigures (i)–(iv) show fluid lamination after passing through 1, 2, 3, and 7 mixing subunits, respectively.


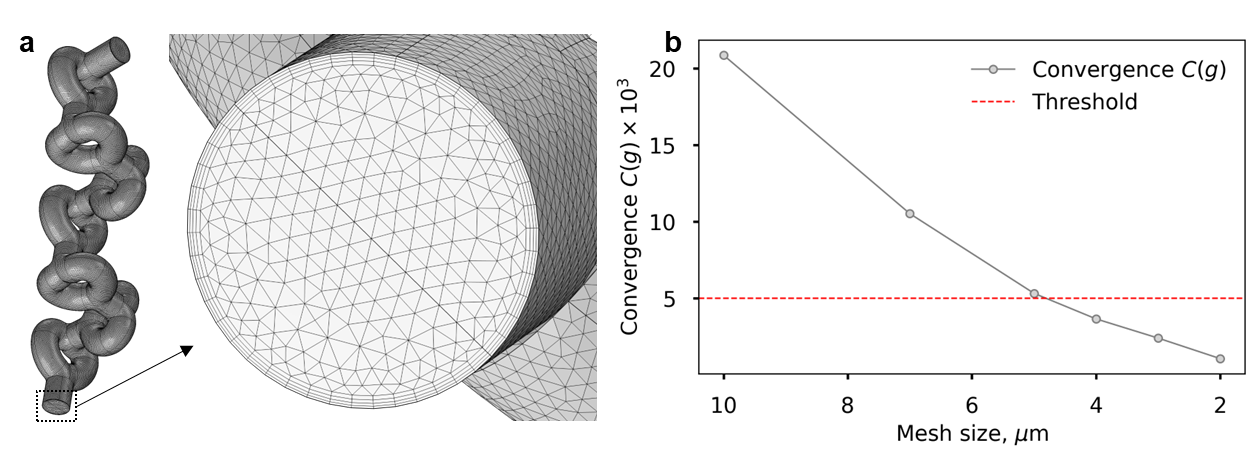


**Fig. S3:** Computational mesh. (a) Overview of the computational mesh with a close-up view of the mesh structure. (b) Mesh convergence study.

**Movie S1:** Assembly process of the 3D-printed micromixer: connection of inlet tubing followed by insertion of the micromixer into the PDMS device.

CAD Files. 3D-printed micromixer design files.

mix_bulk_V3_60.stl – STL file of the micromixer geometry suitable for 3D printing.

mix_bulk_V3_60.asm –Assembly file of the micromixer geometry.

**Table of Contents:**

This 3D-printed modular micromixer interfaces seamlessly with microfluidic devices. Its optimized split-and-recombine geometry enables efficient mixing while minimising internal dead volume and fluidic resistance.


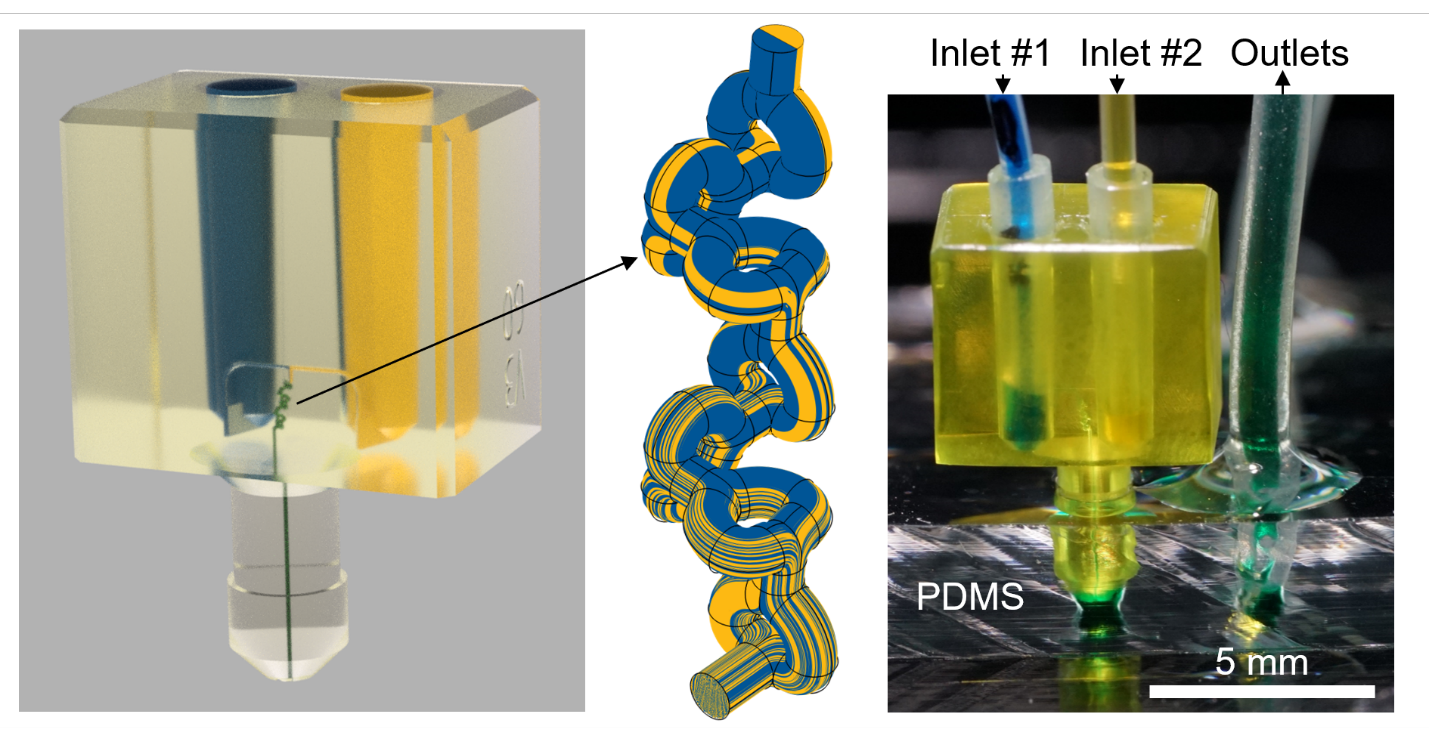

Supplement: Supplementary file 1 — Supporting File 1: advs74647‐sup‐0001‐SuppMat.docx. [file ADVS-13-e10268-s002.docx]
